# Supplementary material for: Influence of pore-confined water on the thermal expansion of a zinc-based metal–organic framework
Source: J Mater Chem C Mater. 2025 Jul 16;13(33):17353–66. doi: 10.1039/d5tc01746g (PMC12302340; doi:10.1039/d5tc01746g)
Supplement: TC-013-D5TC01746G-s001 [file TC-013-D5TC01746G-s001.pdf]

Supplementary Information (SI) for Journal of Materials Chemistry C.
This journal is © The Royal Society of Chemistry 2025

Supporting Information for

Influence of pore-confined water on the thermal expansion of a zinc-based metal-organic framework

Nina Strasser,[a] Benedikt Schrode,[b] Ana Torvisco,[c] Sanjay John,[a] Birgit Kunert,[a] Brigitte Bitschnau,[d] Florian Patrick Lindner,[a] Christian Slugovc,[e] Egbert Zojer*[a] and Roland Resel*[a]

[a] Institute of Solid State Physics, NAWI Graz, Graz University of Technology, Petersgasse 16, 8010 Graz, Austria

[b] Anton Paar GmbH, Anton-Paar-Straße 20, 8054 Graz, Austria

[c] Institute of Inorganic Chemistry, NAWI Graz, Graz University of Technology, Stremayrgasse 9, 8010 Graz, Austria

[d] Institute of Physical and Theoretical Chemistry, NAWI Graz, Graz University of Technology, Stremayrgasse 9, 8010 Graz, Austria

[e] Institute of Chemistry and Technology of Materials, NAWI Graz, Graz University of Technology, Stremayrgasse 9, 8010 Graz, Austria

* Correspondence: egbert.zojer@tugraz.at, roland.resel@tugraz.at

S1. Pores of hydrated and activated GUT-2

The influence of H_2O molecules on the pore structure of hydrated GUT-2 is visualized using a space-filling model, as presented in Fig. S1. By employing a pair of polymer strands and using identical orientations as presented in Fig. 1 (g)-(f) of the manuscript, it is evident that eliminating H_2O molecules leads to the formation of additional pore channels, designated as 'P', which are otherwise inaccessible in the hydrated form.

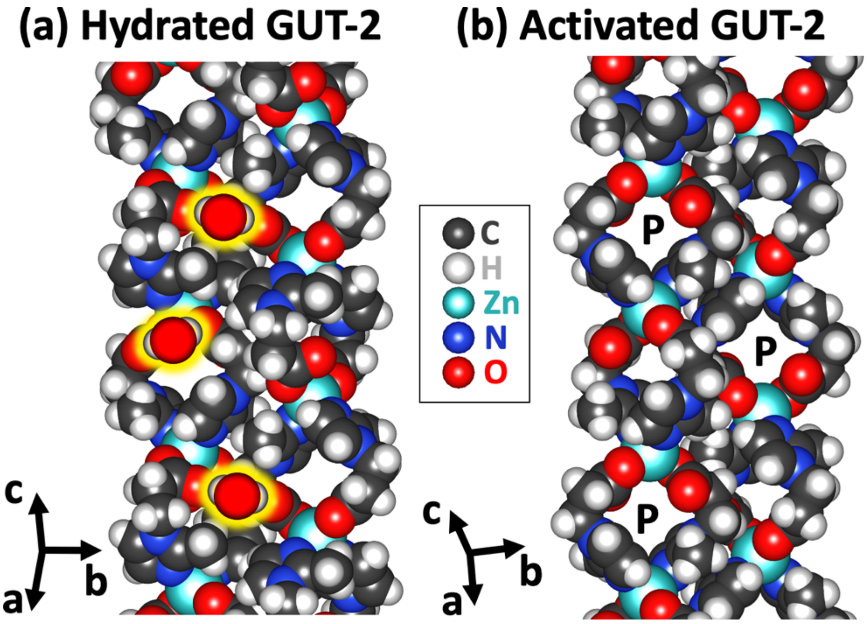

Fig. S1. Space-fill models of two polymer strands of hydrated (**a**) and activated GUT-2 (**b**) where two neighboring polymer strands with the perspective oriented along the axis that connects the centers of two neighboring pores. The H_2O molecules that are present in the hydrated form have been highlighted in yellow and connect two polymer chains. New pores that become available within the activated structure are labelled with 'P'.

Mercury (version: 2024.2.0)[1] revealed that the hydrated form of GUT-2 contains 1.6 % of the unit cell volume (53.54 $Å^3$) as voids capable of accommodating molecules with a maximum probe radius of 1.2 Å. Larger probes yielded no detectable voids, indicating insufficient space for molecules with a larger probe radius than 1.2 Å. Since H_2O is commonly modeled with a probe radius of 1.4 Å in the literature[2] , the available voids in hydrated GUT-2 are thus too small to fit any additional H_2O molecules. Only the smallest possible molecule, H_2, with a probe radius of 1.2 Å, could potentially occupy these voids. A similar situation is observed for the activated form of GUT-2, where 2.0 % of the unit cell volume (32.32 $Å^3$) constitutes of voids of a maximum radius of 1.2 Å.

S2. Detailed discussion of the single crystal diffraction data for hydrated and activated GUT-2

As discussed in the main text, the crystal structure of activated GUT-2 was successfully solved using single crystal X-ray diffraction, while the structure of hydrated GUT-2, already documented in the literature,[3] was reconfirmed. Therefore, Table S1 provides an in-depth comparison of the crystallographic parameters of both GUT-2 forms.

Table S1. Crystal data and structure refinement for the hydrated and activated form of GUT-2 according to the single crystal X-ray diffraction measurements. The parameter Z denotes the number of (chemical) formula units within the unit cell. The value ρ_{calc} represents the calculated crystal density obtained from the unit cell volume and molecular weight. The quantity F(000) represents the total structure factor at zero scattering angle. It corresponds to the sum of the scattering contributions of all atoms in the unit cell and is proportional to the total number of electrons present. The parameter μ is the linear absorption coefficient, describing the extent to which the crystal absorbs X-rays. The R_{int} value quantifies the internal agreement between symmetry-equivalent reflections and serves as an indicator of data consistency. The refinement residuals, R_1 and wR_2, provide a measure of the quality of the structural model, comparing observed and calculated diffraction intensities. The cell parameters (*a*, *b* and *c*) following Niggli convention are, thus, provided in brackets.

	Hydrated GUT-2 (-173°C)[3]	(1) Hydrated GUT-2 (-173°C)	(2) Activated GUT-2 (100°C)
Formula	$C_{14}H_{18}N_4O_4Zn{\cdot}H_2O$	$C_{14}H_{18}N_4O_4Zn{\cdot}H_2O$	$C_{14}H_{18}N_4O_4Zn$
Weight [g/mol]	389.73	389.71	371.69
Temperature [K]	100	100	373
a [Å]	15.1861(13)	15.1721(3)	11.3850(7) [c]
b [Å]	15.0082(13)	14.9839(3)	15.2053(7) [b]
c [Å]	15.0568(13)	15.0445(3)	9.5687(6) [a]
$\alpha = \beta = \gamma$ [°]	90	90	90
Volume [Å^3]	3431.7(5)	3420.17(12)	1656.46(16)
Z	8	8	4
ρ_{calc} [g cm^{-3}]	1.509	1.514	1.490
Crystal system	Orthorhombic	Orthorhombic	Orthorhombic
Space group	Pcca	Pcca	Pccn
Crystal habit	Block, colourless	Block, colourless	Block, colourless
Crystal size [mm^3]	0.05 × 0.05 × 0.04	0.17 × 0.12 × 0.09	0.17 × 0.12 × 0.09
2θ range	2.7–33.1 (Mo K_α)	4.1–76.9 (Cu K_α)	5.8–77.2 (Cu $K\alpha$)
F(000)	1616	1616	768
μ [mm^{-1}]	1.46	2.29	2.29
R_{int}	0.063	0.034	0.10
Independent reflections	2995	3505	1735
No. of parameters	298	223	107
R_1, wR_2 (all reflections)	0.0248, 0.0568	0.0426, 0.0815	0.1049, 0.2518
R_1, wR_2 ($I \geq 2\sigma$)	0.0212, 0.0533	0.0344, 0.0785	0.0839, 0.2335

When evaluating the reliability of a structural model, crystallographers rely on so-called residuals, or R-factors, which quantify the difference between experimental and calculated diffraction data. Overall, lower residuals generally indicate a more reliable and well-refined structural model. These statistical measures help to determine how closely a refined model approximates the actual atomic positions in the crystal. The mathematical foundation of such R-factors is well-established and can be found in textbooks referenced in [4] and [5]. The most common residual, R1, is defined as follows:

$$R_1 = \frac{\sum_{hkl} ||F_{obs}| - |F_{calc}||}{\sum_{hkl} |F_{obs}|}$$

By expressing this value as a percentage, one can easily interpret the agreement between the observed (F_{obs}) and calculated (F_{calc}) structure factors. However, structural refinement also

incorporates weighted contributions, leading to the weighted residual factor, wR_2. This parameter is particularly useful in monitoring the impact of adjustments made during the refinement process, as it reflects the minimized quantity in the least-squares refinement. It is defined in the following way:

$$wR_2 = \sqrt{\frac{\sum_{hkl} w\left(F_{obs}^2 - F_{calc}^2\right)^2}{\sum_{hkl} w\left(F_{obs}^2\right)^2}} \text{with } w = \frac{1}{\sigma^2 F_{obs}^2}$$

The reason for the index 2 in wR2 originates from the fact that the squared structure factors appear in the equation, thereby giving greater weight to more intense reflections. The weighting factor w ensures that reflections with lower uncertainties contribute more significantly to the refinement process. In some refinement programs, additional terms involving adjustable parameters are introduced to further optimize the weighting scheme.

However, what is perhaps more intriguing is the role of temperature, which, despite not being explicitly present in the equation above, has a profound effect on the refinement results. This enhanced movement makes atomic positions less well-defined, introduces greater uncertainty into the structural refinement and increases mosaicity, which is a measurement of the spread of crystal plane orientations. For the hydrated structure measured at -173°C, the ellipsoids are small and well-contained, indicating minimal atomic motion. In contrast, for the activated structure determined at 100°C, they appear significantly larger, illustrating the pronounced increase in atomic vibrations. Consequently, the observed structure factors deviate more strongly from the calculated ones, leading to an increase in R-values. This is precisely why the structure of activated GUT-2, measured at 100°C, exhibits significantly higher R-values than the low-temperature structure of the hydrated form determined at -173°C.

A visual confirmation of this effect is shown in in Fig. S2 (a)-(b) by comparing thermal ellipsoids – graphical representations of atomic displacement within the crystal lattice – of the determined GUT-2 single crystal structures. Mathematically, thermal ellipsoids are described by the atomic displacement parameters, which define the probability density function for atomic positions. The equation governing their shape is given by:

$$\sum_{i=1}^{3} \sum_{j=1}^{3} U_{ij} x_i x_j = 1$$

Here, U_{ij} represents the elements of the anisotropic displacement tensor and x_i and x_j are atomic displacements along the principal crystallographic axes.

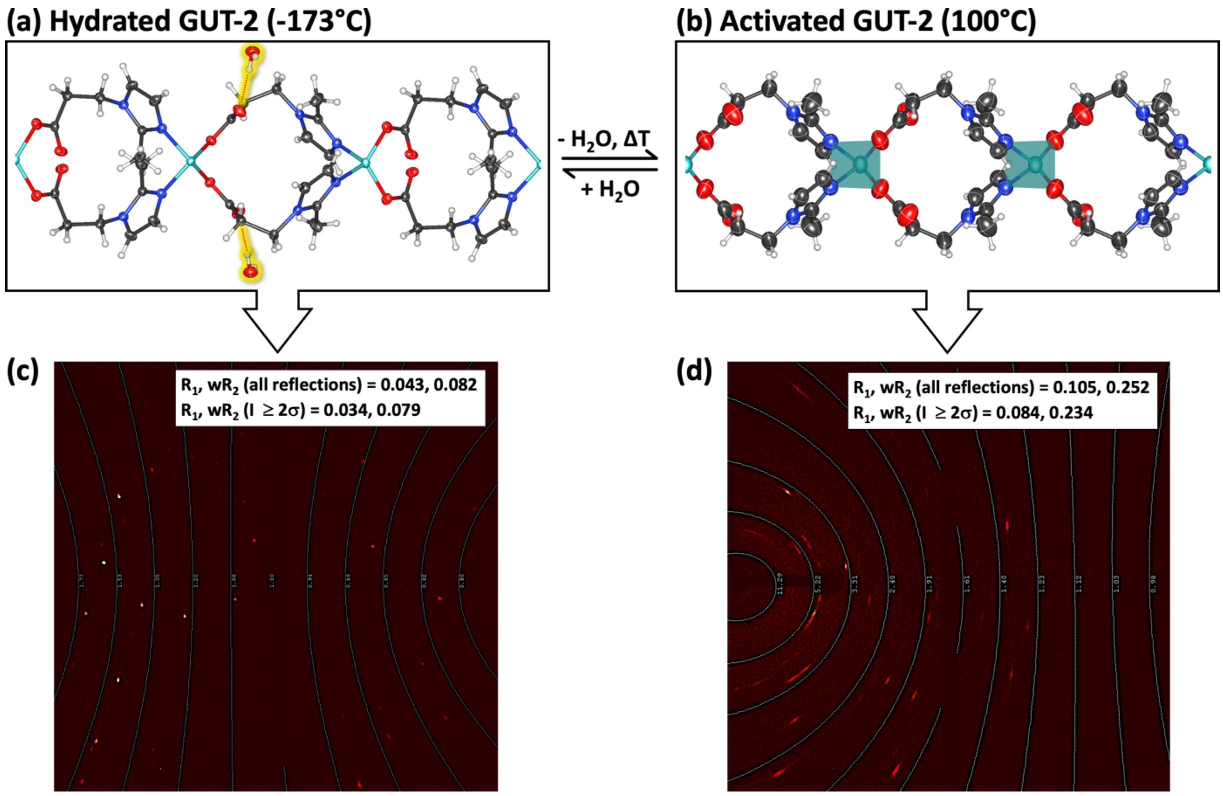

Fig. S2. Single crystal structure solution of a single polymer strand of (**a**) hydrated GUT-2 at -173°C and (**b**) activated GUT-2 at 100°C illustrated with thermal ellipsoids representing the displacement parameters of the atoms (projection along a-axis). These thermal ellipsoids are plotted at the 50 % probability level, meaning that each atom is expected to be located within its ellipsoid with this probability. Hydrogen bonding in the hydrated form is indicated using red dashed lines highlighted in yellow that are connected to the H_2O molecules also highlighted in yellow. Transparent cyan polyhedrals show the tetrahedral bonding situations around the Zn^{2+} ions. The illustrations visualizing the crystal structures are generated using OLEX2 (version 1.5) [6]. On the bottom the corresponding single crystal X-ray diffraction (XRD) pattern for both GUT-2 states are displayed. While the (**c**) hydrated GUT-2 shows sharp, distinct reflections in its XRD pattern, the (**d**) activated GUT-2 shows more diffuse reflections attributed to the elevated temperature at which the measurement has been performed. To enhance the visibility of these effects, the contrast of the original XRD images has been increased by 50 %.

S3. Peak position analysis

Tables S2-S5 contain peak positions for key selected diffraction peaks of hydrated and activated GUT-2 for all four temperature measurement series. For the hydrated crystal domains, the (200) diffraction peak offers a reliable measure of coherent crystal size, while the activated domains are characterized through the (110) diffraction peak. The tabulated results illustrate the gradual evolution of peak widths in both hydrated and activated forms of GUT-2.

Table S2. Peak positions for the (200) diffraction peak (hydrated GUT-2) and the (110) diffraction peak (activated GUT-2) recorded during the first cooling curve.

	Hydrated form, (200) peak	Activated form, (110) peak
Temperature [°C]	Position [°]	Position [°]
25	11.561	12.068
0	11.562	12.061

-50	11.568	12.071
-100	11.572	12.074
-150	11.579	12.090
-190	11.581	12.086

Table S3. Peak positions for the (200) diffraction peak (hydrated GUT-2) and the (110) diffraction peak (activated GUT-2) recorded during the first heating curve.

	Hydrated form, (200) peak	Activated form, (110) peak
Temperature [°C]	Position [°]	Position [°]
-190	11.581	12.086
-150	11.579	12.093
-100	11.576	12.087
-50	11.570	12.089
0	11.568	12.078
50	11.561	12.069
100	11.557	12.075

Table S4. Peak positions for the (110) diffraction peak (activated GUT-2) recorded during the second cooling curve.

	Activated form, (110) peak
Temperature [°C]	Position [°]
100	12.086
50	12.094
0	12.097
-50	12.100
-100	12.107
-150	12.117
-180	12.126

Table S5. Peak positions for the (110) diffraction peak (activated GUT-2) recorded during the second heating curve.

	Activated form, (110) peak
Temperature [°C]	Position [°]
-180	12.126
-150	12.117
-100	12.111
-50	12.104
0	12.103
50	12.094
100	12.088
150	12.081
200	12.069
250	12.057

S4. Instrumental peak broadening

In order to quantify the instrumental broadening effects in PXRD patterns, the standard material LaB_6 was measured using the setup from Anton Paar Ltd (as described in Method Section 3.4). Its diffraction pattern is displayed in Fig. S3 (a), with the peak positions full width at half maximum (FWHM) provided in Table S11. Since the X-ray tube uses Cu as the target material, it generates X-rays with two wavelengths, $K_{\alpha1}$ and $K_{\alpha2}$, which become distinguishable upon zooming into individual peaks, as shown for the (100) peak in Fig. S3 (b).

Given that LaB_6 exhibits its first diffraction peak at approximately 21.4 degrees, and the region of interest for the discussion of the GUT-2 peak widths lies at slightly lower angles, a linear extrapolation was performed on the FWHMs of LaB_6 of the $K_{\alpha1}$ series (see Fig. S3 (c)). In this extrapolation range (from 11.6 degrees to 12.1 degrees), the instrumental FWHM broadening is estimated to be in the range of 0.032 degrees to 0.034 degrees.

Table S6. Peak positions and full widths at half maximum (FWHM) for the K_{α} peak series of LaB_6 in the range from 20 degrees to 80 degrees.

Laue indices	Position [°]	FWHM [°]
(100)	21.360	0.0351
(110)	30.388	0.0350
(111)	37.446	0.0374
(200)	43.511	0.0370
(210)	48.962	0.0377
(211)	53.993	0.0378
(220)	63.224	0.0414
(300)	67.551	0.0410
(310)	71.750	0.0413
(311)	75.847	0.0440

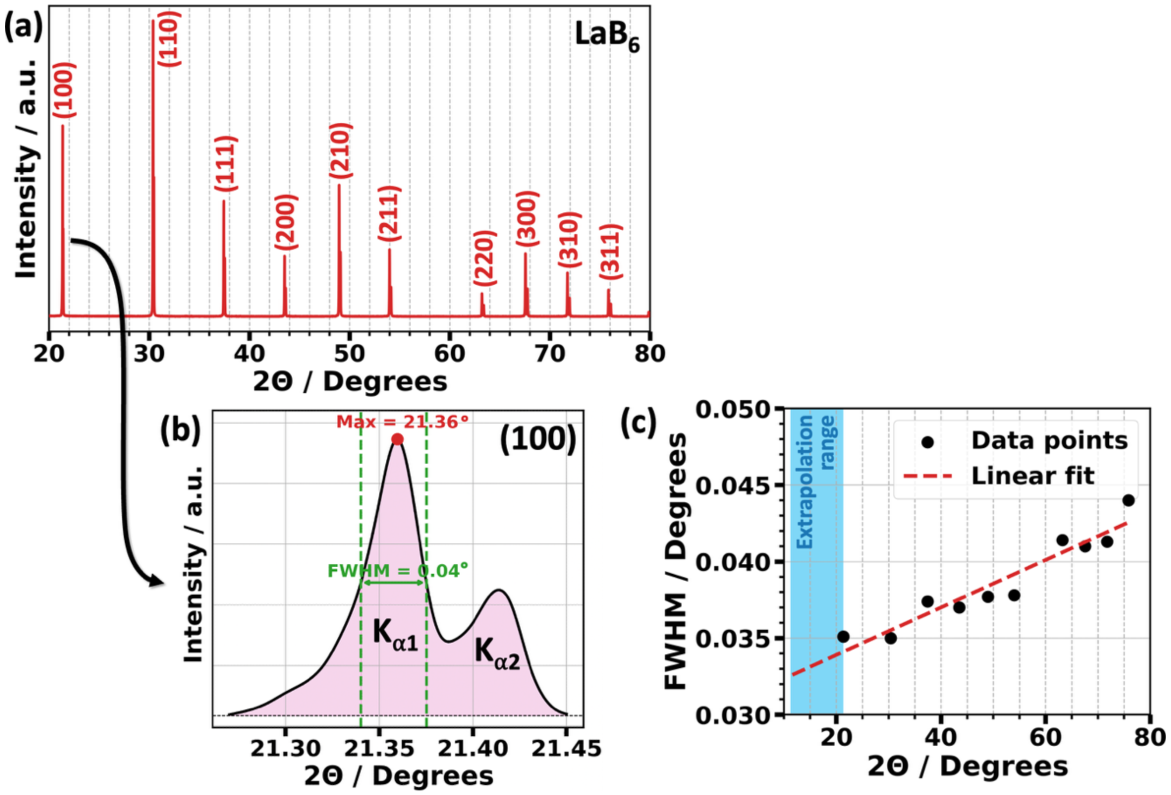

Fig. S3. (**a**) Powder X-ray diffraction pattern of LaB_6 from 20 degrees to 80 degrees including the assignment of Laue indices for all peaks. (**b**) Zoom for the (100) peak of LaB_6 and characterization of the $K_{\alpha 1}$ peak with respect to the position of the maximum and the full width at half maximum (FWHM). Linear fits (red dashed line) for the (**c**) FWHMs (black points) obtained from all $K_{\alpha 1}$ peaks of LaB_6 from 20 degrees to 80 degrees. The range of extrapolation is indicated with a blue rectangle, respectively.

S5. Estimation of crystallite sizes from activated and hydrated GUT-2 powder

From the peak positions reported in Tables S2-S5, the Bragg angles, θ, can be evaluated and from the full width at half maximum (FWHM), denoted as β, it is possible to estimate the size of the crystallites, *D*, from the Scherrer equation[7] in the following way:

$$D = \frac{K\,\lambda}{\beta\cos(\theta)}$$

In this equation, the wave length λ is 1.5418 Å according to the instrument settings and *K*, which is the shape factor, is assumed to be 0.89. The value for *K* stems from the original derivation of the Scherrer equation[7] that assumed crystallites with cubic symmetry. Given that GUT-2 possesses an orthorhombic crystal system with unit cell parameters close to cubic symmetry, this *K* value provides a reasonable assumption for the crystallite size calculations.

The FWHM values are calculated as the peak width at an intensity that corresponds to fifty percent of its highest value Since XRD measurements record a combined signal of both $K\alpha_1$ and $K\alpha_2$ radiation, the overlap of these lines can lead to broadened peaks, affecting the accuracy of the crystal size analysis. Because of that, the Rachinger correction[8] (which is available in the program X'Pert HighScore Plus[9]) was applied before determining the FWHM values to remove the $K\alpha_2$ lines. The estimated crystallite sizes for the hydrated and activated

form of GUT-2 are reported in Tables S7-S10. A detailed discussion of these values can be found in the paper.

Table S7. Full width at half maximum (FWHM) and estimated crystallite sizes using the Scherrer equation[7] for the (200) diffraction peak (hydrated GUT-2) and the (110) diffraction peak (activated GUT-2) recorded during the first cooling curve.

	Hydrated form, (200) peak		Activated form, (110) peak	
Temperature [°C]	FWHM [°]	Crystallite size [nm]	FWHM [°]	Crystallite size [nm]
25	0.0684	116	0.1278	62
0	0.0742	107	0.1288	62
-50	0.0692	114	0.1248	63
-100	0.0736	108	0.1237	64
-150	0.0736	108	0.1237	64
-190	0.0702	113	0.1239	64
	Average size [nm] = 111 $\pm$ 4		Average size [nm] = 63 $\pm$ 1	

Table S8. Full width at half maximum (FWHM) and estimated crystallite sizes using the Scherrer equation[7] for the (200) diffraction peak (hydrated GUT-2) and the (110) diffraction peak (activated GUT-2) recorded during the first heating curve.

	Hydrated form, (200) peak		Activated form, (110) peak	
Temperature [°C]	FWHM [°]	Crystallite size [nm]	FWHM [°]	Crystallite size [nm]
-190	0.0702	113	0.1239	64
-150	0.0735	108	0.1019	78
-100	0.0698	113	0.1191	67
-50	0.0687	115	0.1190	67
0	0.0705	112	0.1209	66
50	0.0717	111	0.1151	69
100	0.0754	105	0.1084	73
	Average size [nm] = 111 $\pm$ 3		Average size [nm] = 69 $\pm$ 5	

Table S9. Full width at half maximum (FWHM) and estimated crystallite sizes using the Scherrer equation[7] for the (110) diffraction peak (activated GUT-2) recorded during the second cooling curve.

	Activated form, (110) peak	
Temperature [°C]	FWHM [°]	Crystallite size [nm]
100	0.1084	73
50	0.1016	78
0	0.1035	76
-50	0.1039	76
-100	0.1045	76
-150	0.1050	75
-180	0.1046	76
	Average size [nm] = 76 $\pm$ 2	

Table S10. Full width at half maximum (FWHM) and estimated crystallite sizes using the Scherrer equation[7] for the (110) diffraction peak (activated GUT-2) recorded during the second heating curve.

	Activated form, (110) peak	
Temperature [°C]	FWHM [°]	Crystallite size [nm]
-180	0.1046	76
-150	0.1050	75

-100	0.1041	76
-50	0.1015	78
0	0.1040	76
50	0.1040	76
100	0.1042	76
150	0.1022	78
200	0.1030	77
250	0.1019	78
	Average size [nm] = 77 ± 1	

S6. Temperature-dependent cell parameters of hydrated and activated GUT-2 obtained from Rietveld refinements

To determine temperature-dependent cell parameters and derive direction-specific thermal expansion coefficients, Rietveld refinements were performed. The outcomes of these Rietveld refinements for the four sections of the temperature cycle shown in Fig. 2 (a) are detailed in Fig. S4–S9. The data reveal that, for the hydrated form of GUT-2 in C1 and H1, not all peaks are accurately captured using only the hydrated structure model. In fact, it turned out that incorporating also the activated structure model of GUT-2 in the refinement process is necessary to account for all experimental peaks (for details see main manuscript).

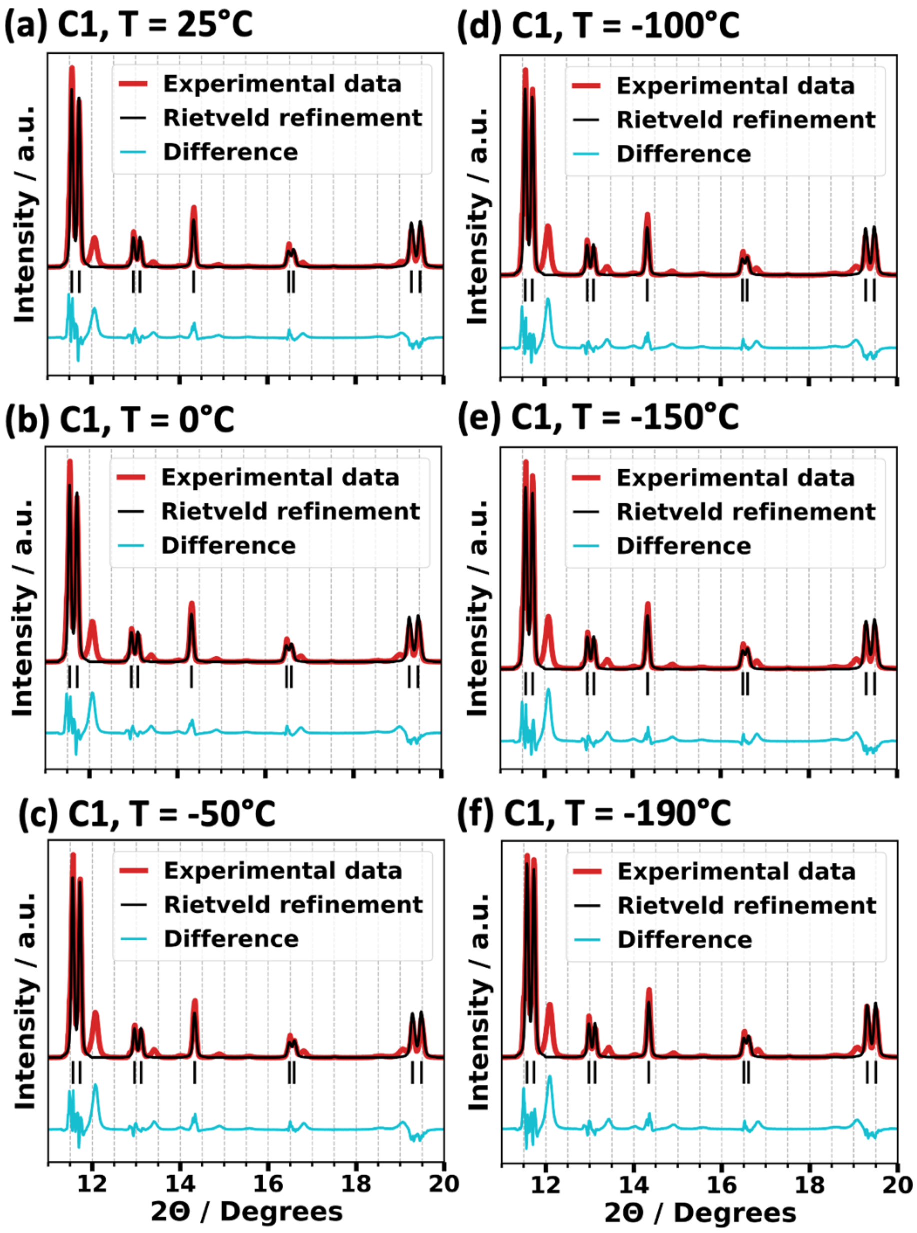

Fig. S4. Overlay of the temperature-dependent powder X-ray diffraction pattern (red curves) and the Rietveld refinements (black curves) based on the hydrated form of GUT-2 for the first cooling cycle (C1). The calculated Bragg peaks are shown as vertical lines. The difference between the experimental data and the Rietveld refinement is shown as a blue curve below in each of the subplots.

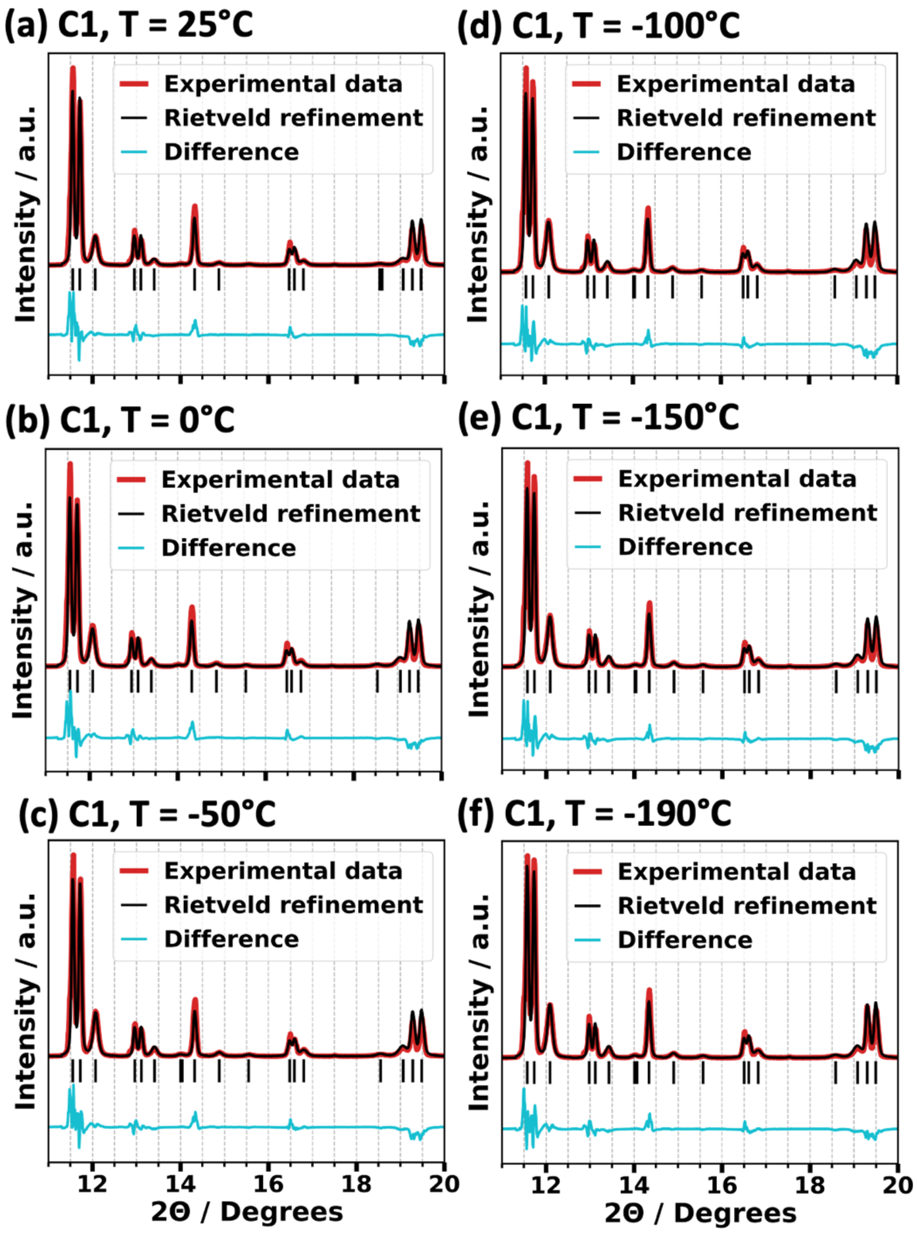

Fig. S5. Overlay of the temperature-dependent powder X-ray diffraction pattern (red curves) and the Rietveld refinements (black curves) based on the hydrated and activated form of GUT-2 for the first cooling cycle (C1). The calculated Bragg peaks are shown as vertical lines. The difference between the experimental data and the Rietveld refinement is shown as a blue curve below in each of the subplots.

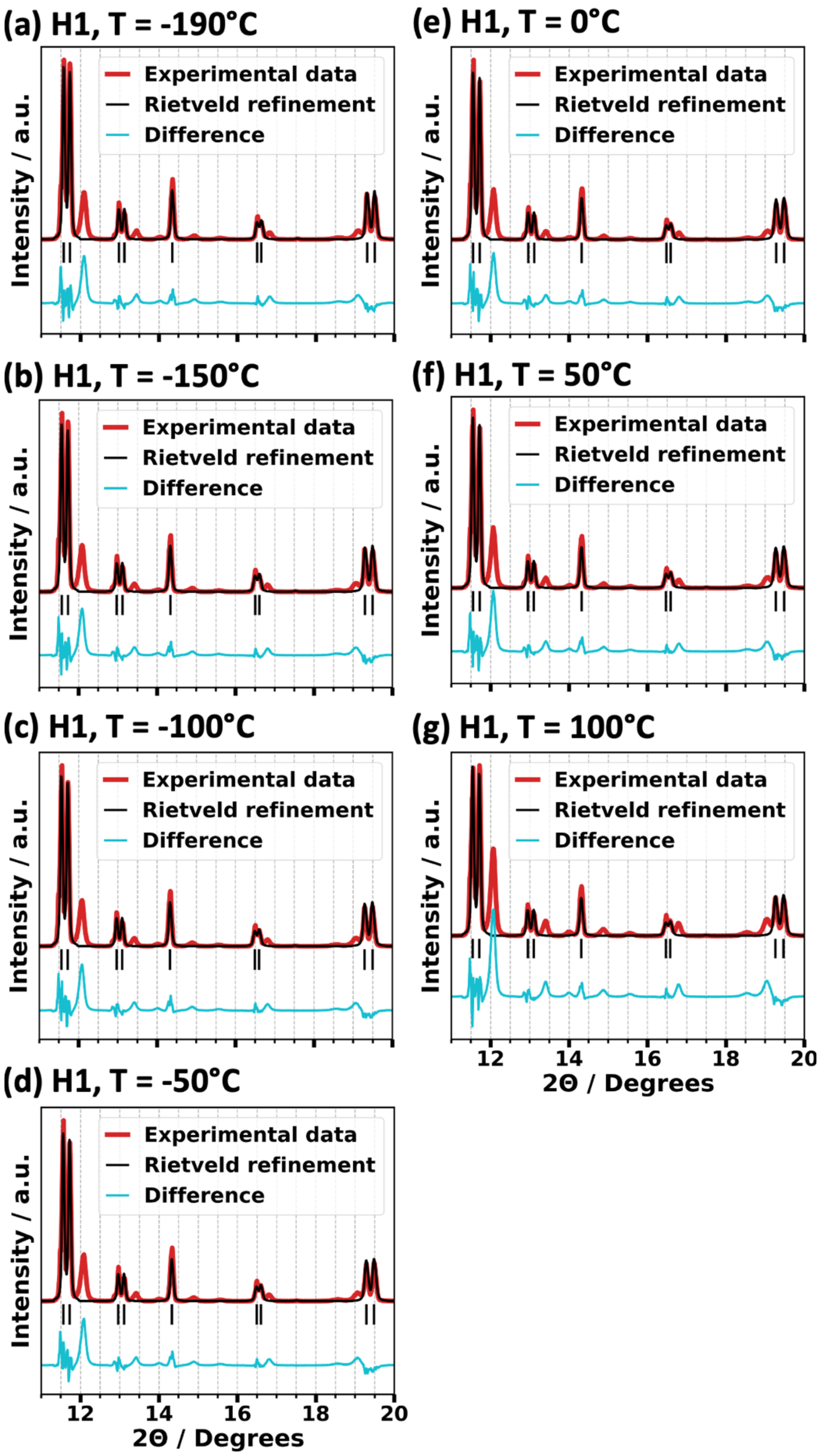

Fig. S6. Overlay of the temperature-dependent powder X-ray diffraction pattern (red curves) and the Rietveld refinements (black curves) based on the hydrated form of GUT-2 for the first heating cycle (H1). The calculated Bragg peaks are shown as vertical lines. The difference between the experimental data and the Rietveld refinement is shown as a blue curve below in each of the subplots.

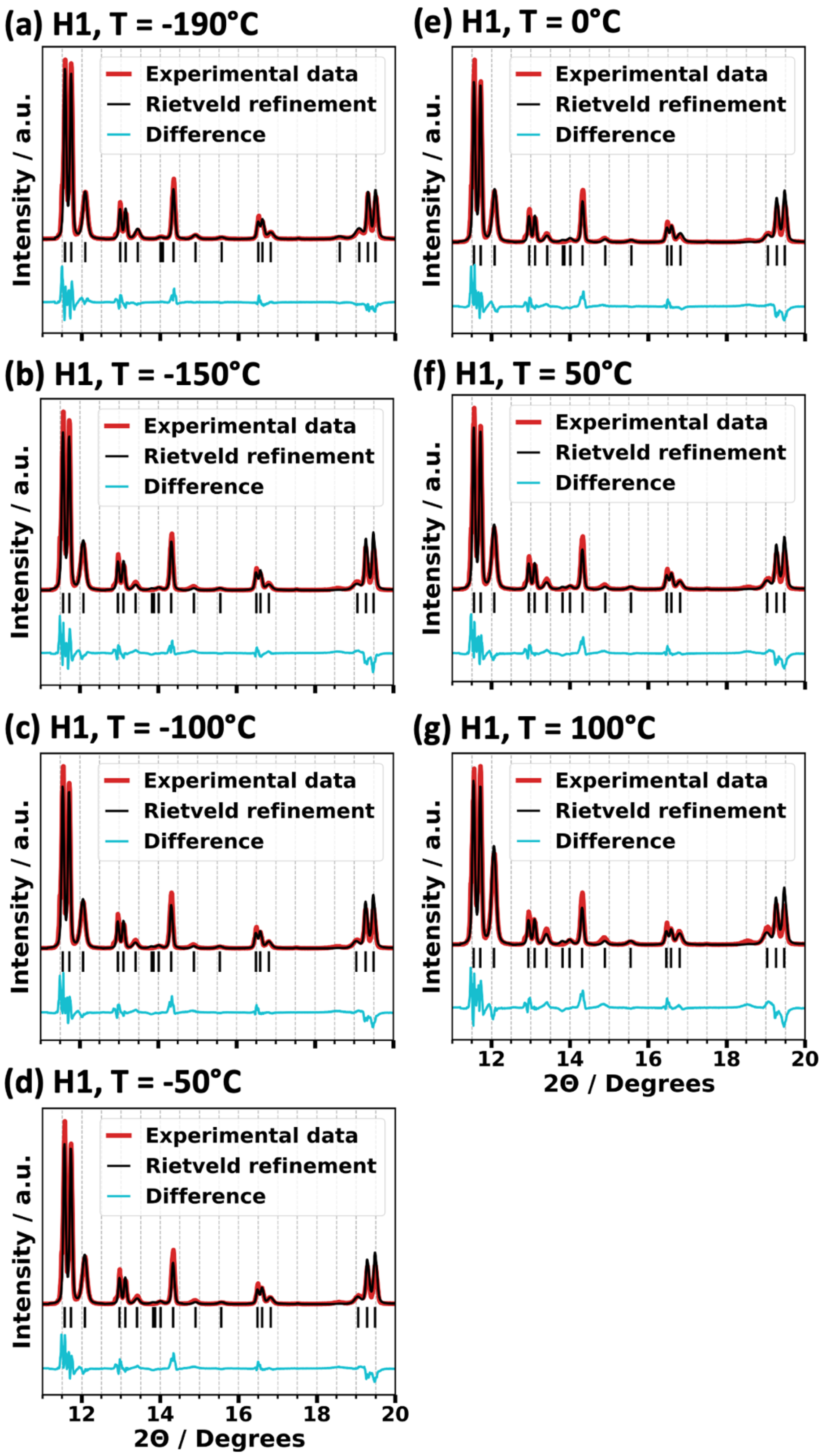

Fig. S7. Overlay of the temperature-dependent powder X-ray diffraction pattern (red curves) and the Rietveld refinements (black curves) based on the hydrated and activated form of GUT-2 for the first heating cycle (H1). The calculated Bragg peaks are shown as vertical lines. The difference between the experimental data and the Rietveld refinement is shown as a blue curve below in each of the subplots.

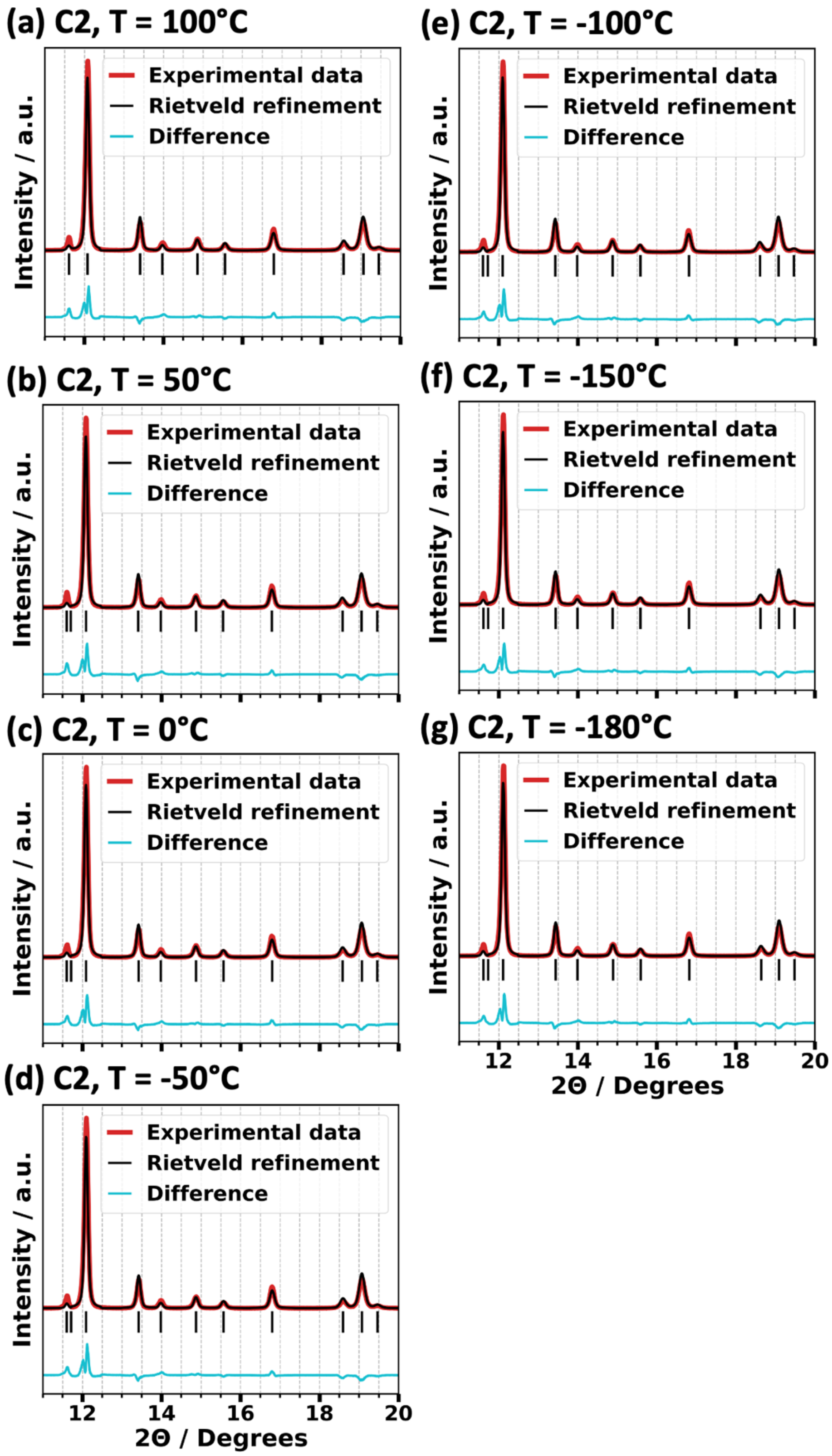

Fig. S8. Overlay of the temperature-dependent powder X-ray diffraction pattern (red curves) and the Rietveld refinements (black curves) based on the activated form of GUT-2 for the second cooling cycle (C2). The calculated Bragg peaks are shown as vertical lines. The difference between the experimental data and the Rietveld refinement is shown as a blue curve below in each of the subplots.

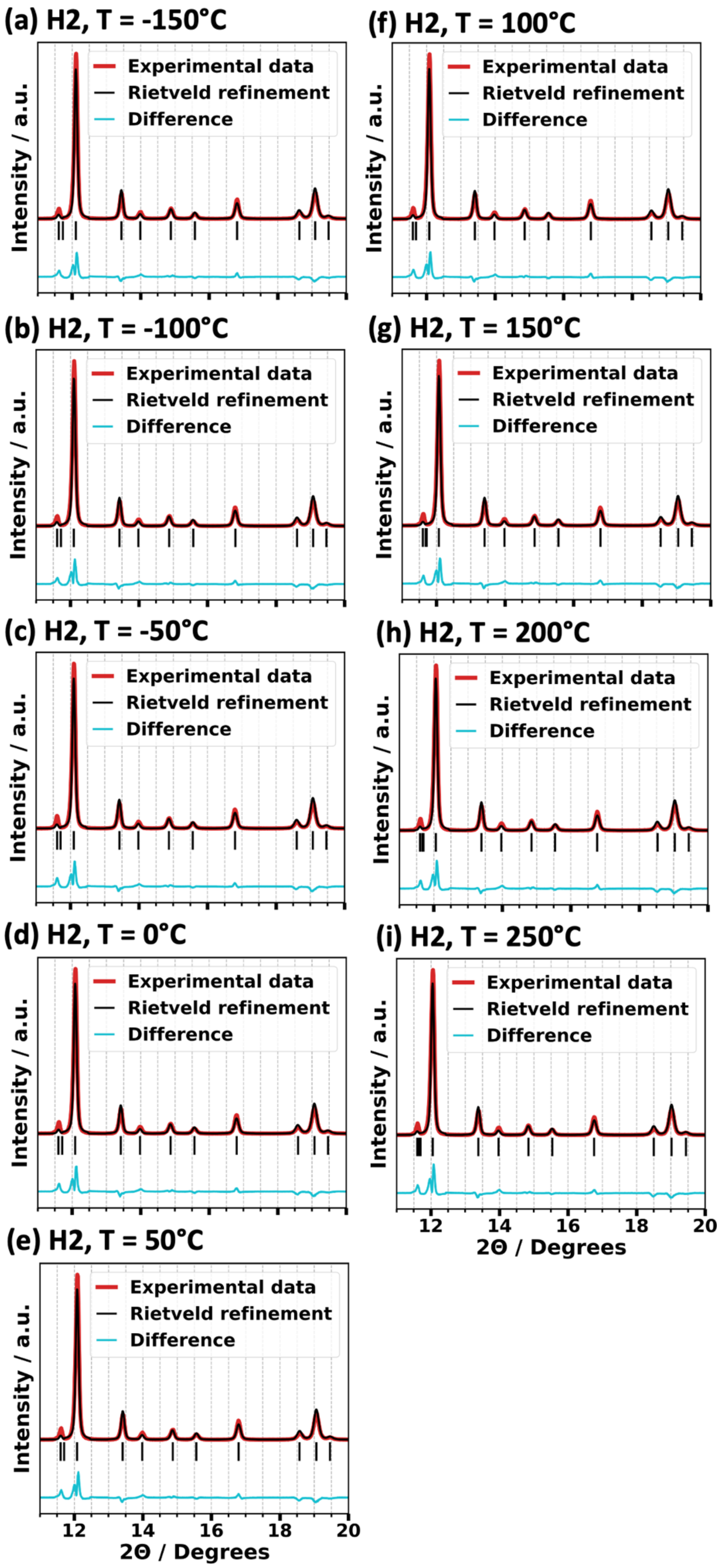

Fig. S9. Overlay of the temperature-dependent powder X-ray diffraction pattern (red curves) and the Rietveld refinements (black curves) based on the activated form of GUT-2 for the second heating cycle (H2). The calculated Bragg peaks are shown as vertical lines. The difference between the experimental data and the Rietveld refinement is shown as a blue curve below in each of the subplots.

Three statistical parameters, R_p, R_{wp}, and R_{exp} are commonly used in Rietveld refinement to measure how well the observed diffraction pattern matches the calculated pattern from the model, allowing for an evaluation of the quality of the refinement. R_p is called the R-pattern or profile residual and compares the difference between the observed intensity y_i and the calculated intensity y_c for each data point in the diffraction pattern. The closer R_p is to zero, the better the fit.

$$R_p = \frac{\sum |y_i - y_c|}{\sum y_i}$$

R_{wp} is a weighted version of R_p and is, therefore, called the weighted profile residual. It takes into account the uncertainty in the observed intensities (I_{obs}), typically giving more weight w_i to data points with higher intensities (lower noise). Here, σ is the standard deviation of the observed intensity, which is typically determined from counting statistics. In diffraction experiments, the uncertainty in measured (observed) intensities follows Poisson statistics.

$$R_{wp} = \sqrt{\frac{\sum w_i (y_i - y_c)^2}{\sum w_i {y_i}^2}} \text{ with } w_i = \frac{1}{\sigma_i^2} \text{ where } \sigma(I_{obs}) = \sqrt{I_{obs}}$$

Finally, the expected R-factor, R_{exp}, provides a measure of how good the fit could be based purely on the level of noise. It depends on the number of data points N and on the number of parameters that are refined denoted with P.

$$R_{exp} = \sqrt{\frac{(N - P)}{\sum w_i y_i^2}}$$

A summary of the three statistical parameters R_p, R_{wp} and R_{exp} alongside with the temperature-dependent cell parameters *a*, *b* and *c* together with the volume *V* can be found in Tables S11-S14 for all measured points on both cooling and heating curves.

Table S11. Temperature-dependent cell parameters of hydrated GUT-2 for the first cooling curve. R_p (R-pattern), R_{wp} (R-weighted pattern) and R_{exp} (R-expected) are the standard reliability factors used in Rietveld refinement to assess the quality of the fit between the observed and calculated diffraction patterns. R_p^* is obtained using both the activated and hydrated form simultaneously for the Rietveld refinement.

T [°C]	a [Å]	b [Å]	c [Å]	V [Å^3]	R_p^* [%]	R_p [%]	R_{wp} [%]	R_{exp} [%]
25	15.2901	15.0972	15.0728	3479.36	18.85	28.03	38.33	2.23
0	15.2895	15.0980	15.0725	3479.34	18.36	29.96	41.76	2.21
-50	15.2874	15.0985	15.0713	3478.71	18.46	31.18	43.79	2.19
-100	15.2792	15.0920	15.0676	3474.52	18.17	31.58	44.43	2.19
-150	15.2711	15.0848	15.0624	3469.80	18.17	36.09	51.49	2.20
-190	15.2601	15.0790	15.0560	3464.36	16.33	30.33	43.76	2.20

Table S12. Temperature-dependent cell parameters of hydrated GUT-2 for the first heating curve. R_p (R-pattern), R_{wp} (R-weighted pattern) and R_{exp} (R-expected) are the standard reliability factors used in Rietveld refinement to assess the quality of the fit between the observed and calculated diffraction

patterns. R_p^* is obtained using both the activated and hydrated form simultaneously for the Rietveld refinement.

T [°C]	a [Å]	b [Å]	c [Å]	V [Å^3]	R_p^* [%]	R_p [%]	R_{wp} [%]	R_{exp} [%]
-190	15.2601	15.0790	15.0560	3464.36	16.33	30.33	43.76	2.20
-150	15.2638	15.0816	15.0574	3466.25	18.81	30.46	43.81	2.20
-100	15.2718	15.0895	15.0628	3471.13	18.82	30.30	43.73	2.18
-50	15.2766	15.0933	15.0645	3473.49	19.01	30.64	44.05	2.18
0	15.2826	15.0969	15.0665	3476.13	20.14	31.64	45.02	2.18
50	15.2903	15.1036	15.0701	3480.28	20.02	33.95	47.21	2.18
100	15.2947	15.1093	15.0721	3483.05	21.45	39.39	52.96	2.18

Table S13. Temperature-dependent cell parameters of activated GUT-2 for the second cooling curve R_p (R-pattern), R_{wp} (R-weighted pattern) and R_{exp} (R-expected) are the standard reliability factors used in Rietveld refinement to assess the quality of the fit between the observed and calculated diffraction patterns.

T [°C]	a [Å]	b [Å]	c [Å]	V [Å^3]	R_p [%]	R_{wp} [%]	R_{exp} [%]
100	11.3682	15.2203	9.5433	1651.17	17.62	21.76	2.16
50	11.3686	15.2186	9.5407	1649.67	17.38	22.96	2.16
0	11.3654	15.2181	9.5357	1649.29	17.90	22.15	2.16
-50	11.3631	15.2174	9.5311	1648.09	17.70	22.00	2.16
-100	11.3596	15.2166	9.5247	1646.37	17.48	21.77	2.16
-150	11.3538	15.2126	9.5145	1643.35	17.44	21.67	2.17
-180	11.3503	15.2118	9.5087	1641.77	17.22	21.37	2.17

Table S14. Temperature-dependent cell parameters of activated GUT-2 for the second heating curve. R_p (R-pattern), R_{wp} (R-weighted pattern) and R_{exp} (R-expected) are the standard reliability factors used in Rietveld refinement to assess the quality of the fit between the observed and calculated diffraction patterns.

T [°C]	a [Å]	b [Å]	c [Å]	V [Å^3]	R_p [%]	R_{wp} [%]	R_{exp} [%]
-180	11.3503	15.2118	9.5087	1641.77	17.22	21.37	2.17
-150	11.3548	15.2156	9.5150	1643.91	17.24	21.39	2.17
-100	11.3594	15.2185	9.5232	1646.31	17.40	21.56	2.17
-50	11.3624	15.2186	9.5288	1647.71	17.64	22.16	2.16
0	11.3658	15.2202	9.5363	1649.68	17.93	22.50	2.16
50	11.3684	15.2198	9.5418	1650.96	17.60	22.13	2.16
100	11.3726	15.2217	9.5486	1652.98	17.82	22.37	2.16
150	11.3764	15.2228	9.5585	1655.35	17.90	22.53	2.16
200	11.3840	15.2240	9.5672	1658.09	18.22	22.86	2.16
250	11.3912	15.2261	9.5803	1661.63	18.35	23.14	2.15

S7. Test runs causing partially activation of hydrated GUT-2

Before recording the powder X-ray diffraction (PXRD) pattern of the actual cooling curve C1, reported in the main manuscript, two preliminary test runs were conducted for the first cooling cycle, whose temperature-dependent PXRD pattern are given in Fig. S10 (a)-(b). Phase

quantification through Rietveld refinements shown in Fig. S11 (a)-(b) indicates a partial activation of the powder sample during these preliminary tests. Specifically, each time the sample was cooled from 25°C to 0°C, approximately 10 % activation was observed, which we attribute to the fact that the experiments were performed in vacuum. Consequently, in the temperature-dependent PXRD pattern, features of the activated GUT-2 form are present. This is seen, for example, for the at 12.1° and indicates an activation level of around 30 %. As shown in Fig. S11 (a), phase quantification of the first heating curve reveals that the sample starts at 30 % activation. Significant further activation of the GUT-2 powder only begins after approximately two hours, when the temperature reaches around 50°C.

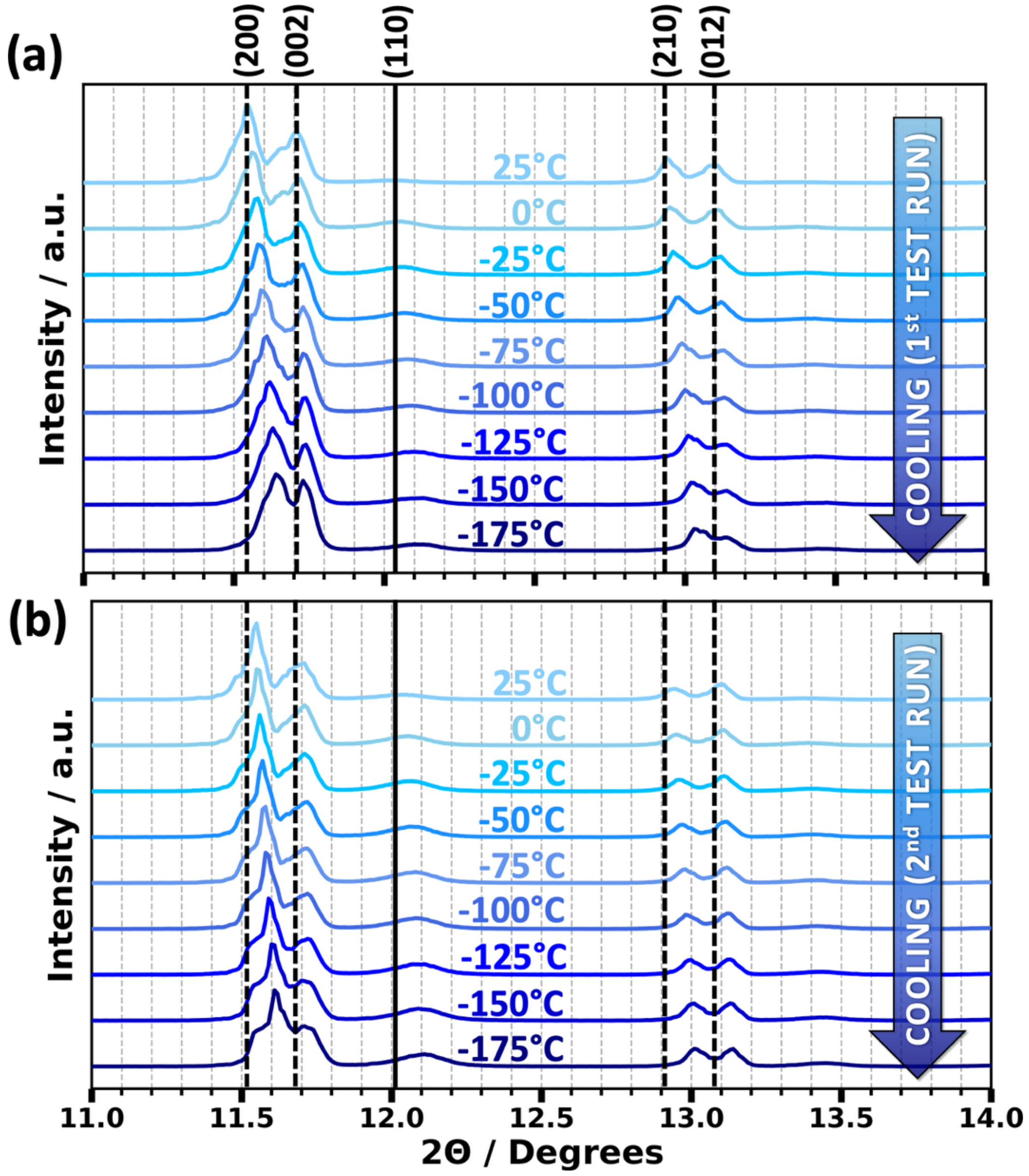

Fig. S10. (**a**)-(**b**) Temperature-dependent powder X-ray diffraction pattern in the range from 11 to 14 degrees for two cooling cycle test runs of GUT-2. Peaks associated with hydrated GUT-2 are highlighted by dashed vertical lines, whereas solid vertical lines indicate the peaks of the activated form.

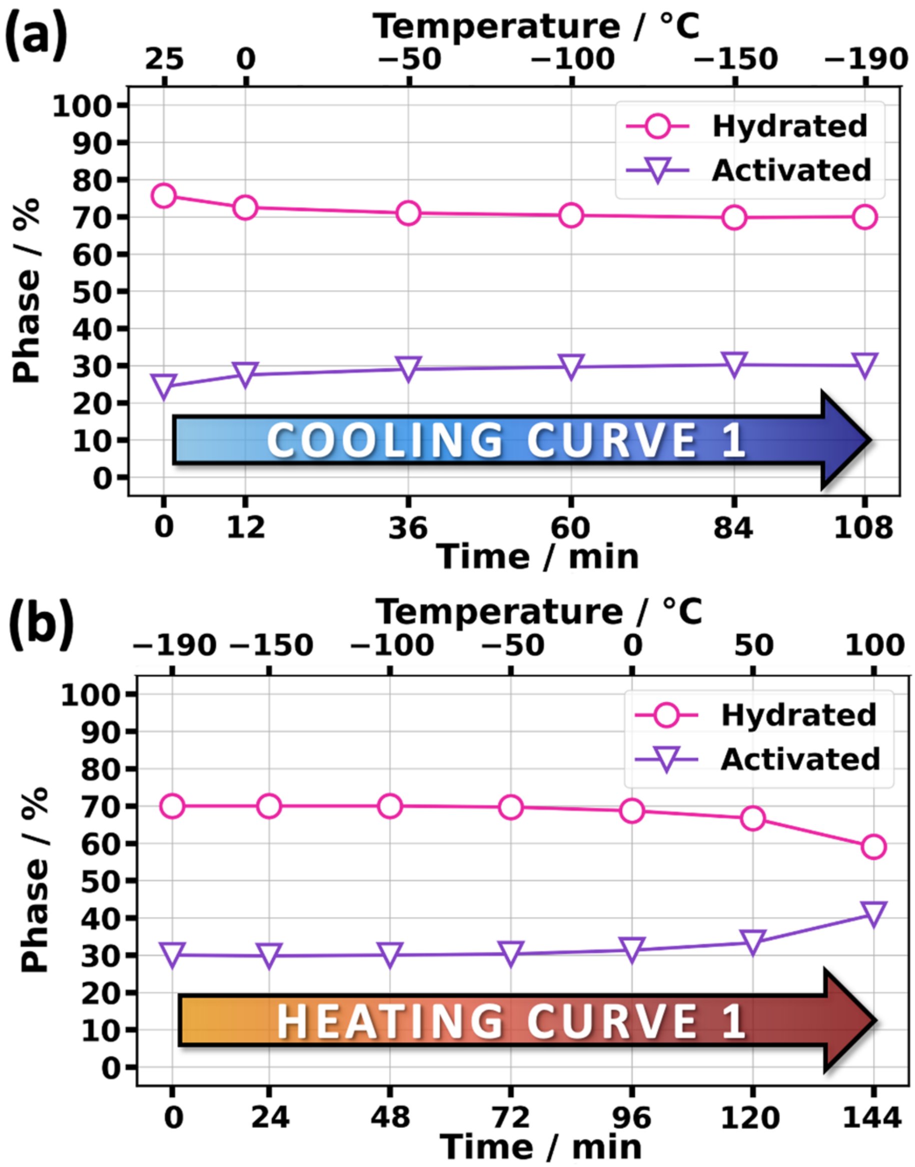

Fig. S11. Phase quantifications for the first cooling (**a**) and first heating curve (**b**) with respect to temperature based on Rietveld refinements. The data points for the amount of hydrated form are shown as pink circles (○), while the data points for the percentage of activated phase are represented as purple, inverted triangles (∇).

S8. Thermal expansion and bonding strengths

The thermal expansion of materials is fundamentally linked to the type of interatomic interactions. A useful way to describe these interactions is to perform the Taylor expansion of a general anharmonic potential around its minimum (as shown for example in the solid state physics textbooks by Gross and Marx[10] or by Kittel[11]), given by:

$$\mathrm{U(x) = cx^2 - gx^3}$$

where *x* represents the atomic displacement and the coefficients *c* and *g* determine the curvature and the asymmetry of the potential well. This polynomial approximation captures

the essential anharmonic effects. Unlike the Morse potential, which has an exponential decay for large bond lengths, this polynomial expansion does not describe any bond breaking.

To derive the thermal expansion from the potential energy function *U(x)*, we can use the Boltzmann probability distribution to calculate the expectation value of the atomic displacement, denoted as $\langle x \rangle$ with $\beta = 1/k_BT$ (k_B refers to as the Boltzmann constant, *T* is the temperature) and expanding $e^{-\beta f(x)}$ using a Taylor series around the harmonic term[11]:

$$\langle x \rangle = \frac{\int_{-\infty}^{\infty} x e^{-\beta U(x)} dx}{\int_{-\infty}^{\infty} e^{-\beta U(x)} dx} \cong \frac{\int_{-\infty}^{\infty} e^{-\beta c x^2} (x + \beta g x^4) dx}{\int_{-\infty}^{\infty} e^{-\beta c x^2} dx} = \frac{\frac{3\pi^{1/2}}{4} \frac{g}{c^{5/2}} \beta^{-3/2}}{\left(\frac{\pi}{\beta c}\right)^{1/2}} = \frac{3g}{4c^2} k_B T$$

From this result, we see that the cubic and quartic terms of U(x) introduce thermal expansion. This means that for a harmonic oscillator (g = 0), $\langle x \rangle$ remains constant and thus this model is unable to describe any thermal expansion. In contrast, as soon as anharmonic terms ($g \neq 0$) are present, $\langle x \rangle$ changes with temperature, leading to thermal expansion. Moreover, the coefficient c defines the curvature at the minimum of the potential well and is directly linked to the bond stiffness. The stronger the chemical bond, the higher c becomes the smaller thermal movement of the atoms. Therefore, as long as g does not increase at least with c-squared, a more strongly bonding potential is expected to result in a reduced thermal expansion.

S9. Kinetics of H_2O adsorption/desorption

In the course of experiments, we investigated the kinetics of H_2O adsorption and desorption of GUT-2 in greater detail. The temperature-dependent PXRD patterns and corresponding phase quantifications of these kinetic studies are displayed in Fig. S12. As outlined in the manuscript, hydrated GUT-2 undergoes activation relatively easily: When the sample is heated to 90°C, full activation occurs within approximately 30 minutes, as evidenced by a pronounced alteration in the PXRD pattern. In a second experiment, heating was limited to 50°C, leading to full activation after roughly 6 hours.

We also examined the rehydration process (Fig. S12 (c)) by exposing activated GUT-2 to standard laboratory conditions (30 % relative humidity). Here, H_2O adsorption occurs much more slowly, beginning after several hours with a decrease in the 12.1 degrees peak intensity in the PXRD pattern. Complete rehydration, however, requires at least two days (see Fig. S12 (f)), though the process of H_2O ad- and desorption remains fully reversible keeping the framework intact.

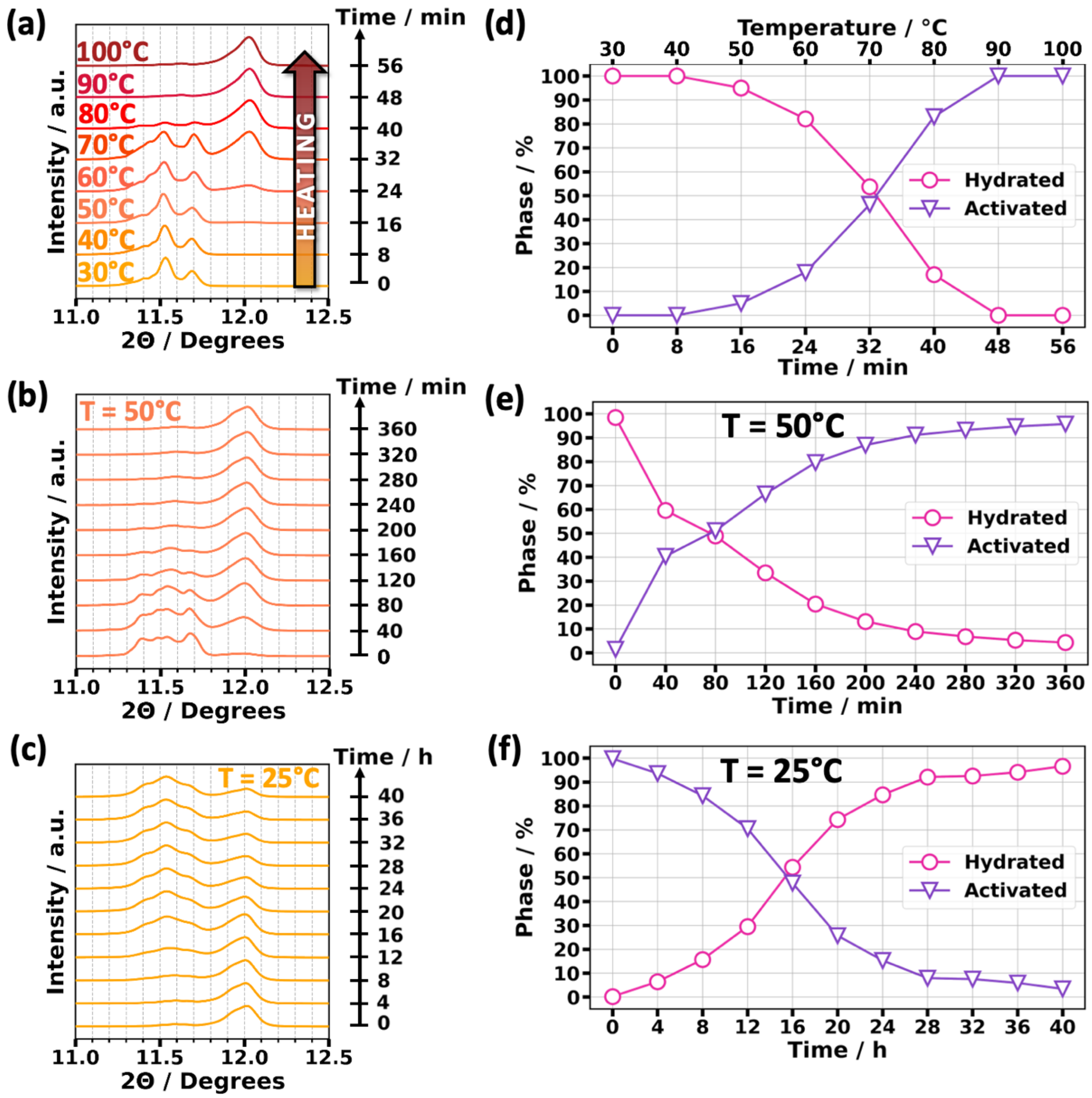

Fig. S12. Experimental powder X-ray diffraction pattern (PXRD) for the activation of GUT-2 using a steady heating ramp from 30°C to 100°C over approximately 1 h (**a**) and at a constant temperature of 50°C for 6 h (**b**) in the range of 11.0 to 12.5 degrees. (**c**) Experimental PXRD pattern for the hydration of GUT-2 at RT over 40 h in the range of 11.0 to 12.5 degrees. Panels (**d**)-(**f**) show the corresponding phase quantifications for the activated and hydrated form of GUT-2 that were determined via Rietveld refinements for different temperatures. Here, the data points for the hydrated form are shown as pink circles (○), while the data points for the activated form are represented as purple, inverted triangles (∇).

S10. Mechanical properties of GUT-2

For infinitesimal deformation strains the crystal energy ε can be expressed by a quadratic form of the elastic tensor:[12]

$$E = E_0 + \frac{V_0}{2}\sum_{i,j} C_{ij}\varepsilon_i\varepsilon_j + O(\varepsilon^3)$$

A crystal structure is said to be mechanically stable if – in addition to the structure having no negative phonon modes (dynamical stability) – the energy contribution of the quadratic form is always positive. This is mathematically equivalent to the condition that the matrix C is positive definite, i.e., possessing no negative eigenvalues.

As discussed in greater detail in the Supporting Information of Ref.[13] elastic constants are difficult to obtain using density functional theory (DFT). Here, we employ the CRYSTAL23 code[14], which thanks to the use of numerically efficient atom-centered basis sets allows for the calculation of the fully ion-relaxed elastic tensor elements. Regarding the basis sets we used Zn[8-64111-41G(f)][15,16] for the metallic node and C/N/H/O[6-311G(d,p)][16,17] for the linker atoms. Similar to the calculation of the elastic tensor elements for the hydrated structure of GUT-2 in Ref.[13] we set the convergence criteria TOLDEG and TOLDEX to 0.0002 and 0.0004, respectively, and we used the PBE functional[18] in combination with the D3 van der Waals correction.[19] A strain step of 0.05 Å was applied. The computed elastic tensors are provided in Table S15.

Table S15. Calculated elastic tensors for hydrated and activated form of GUT-2 using CRYSTAL23.

Hydrated GUT-2 (taken from Ref.[13])	Activated GUT-2
$C_{DFT} = \begin{pmatrix} 19.8 & 12.4 & 8.7 & 0 & 0 & 0 \\ 12.4 & 22.8 & 14.3 & 0 & 0 & 0 \\ 8.7 & 14.3 & 21.4 & 0 & 0 & 0 \\ 0 & 0 & 0 & 4.4 & 0 & 0 \\ 0 & 0 & 0 & 0 & 5.0 & 0 \\ 0 & 0 & 0 & 0 & 0 & 5.2 \end{pmatrix}$ GPa	$C_{DFT} = \begin{pmatrix} 8.5 & 6.7 & 9.6 & 0 & 0 & 0 \\ 6.7 & 12.3 & 8.7 & 0 & 0 & 0 \\ 9.6 & 8.7 & 19.0 & 0 & 0 & 0 \\ 0 & 0 & 0 & 4.8 & 0 & 0 \\ 0 & 0 & 0 & 0 & 3.5 & 0 \\ 0 & 0 & 0 & 0 & 0 & 4.3 \end{pmatrix}$ GPa

Although there are three trivial eigenvalues of the matrix C in case of orthorhombic crystal symmetry that need to be positive, namely C_{44}, C_{55} and C_{66}, it is not possible to give a closed form expression for the Born stability criterion[12] for the remaining three eigenvalues. Hence, we have to calculate them brute-force and check their positivity. All six eigenvalues of the elastic tenors for both structures are listed in Table S16. As can be seen from this table all eigenvalues are strictly positive, hence the Born stability criterion for mechanical stability is fulfilled for both structures.

Table S16. Eigenvalues of the elastic tensors for hydrated and activated form of GUT-2 using CRYSTAL23.

Eigenvalue [GPa]	Hydrated GUT-2	Activated GUT-2
λ_1	4.4	2.5
λ_2	4.9	3.5
λ_3	5.2	4.3
λ_4	6.8	4.8
λ_5	12.0	6.4
λ_6	45.3	31.0

S11. Recorded Temperature Profile via Temperature Sensor

The temperature program, as recorded by the temperature sensor, is presented in Figure S13. In passing, we note that in this temperature graph, the rapid increase in temperature after around 20.5 h is caused due to a disruption in the cooling process, likely resulting from moisture entering the liquid nitrogen supply line during refilling at 25°C. This moisture subsequently froze, reducing the cooling efficiency. In response, the cooling was halted and a new experiment was launched targeting -180°C as the minimum temperature. Unfortunately, during this transition, the sample warmed up to around -90°C for a short period of time.

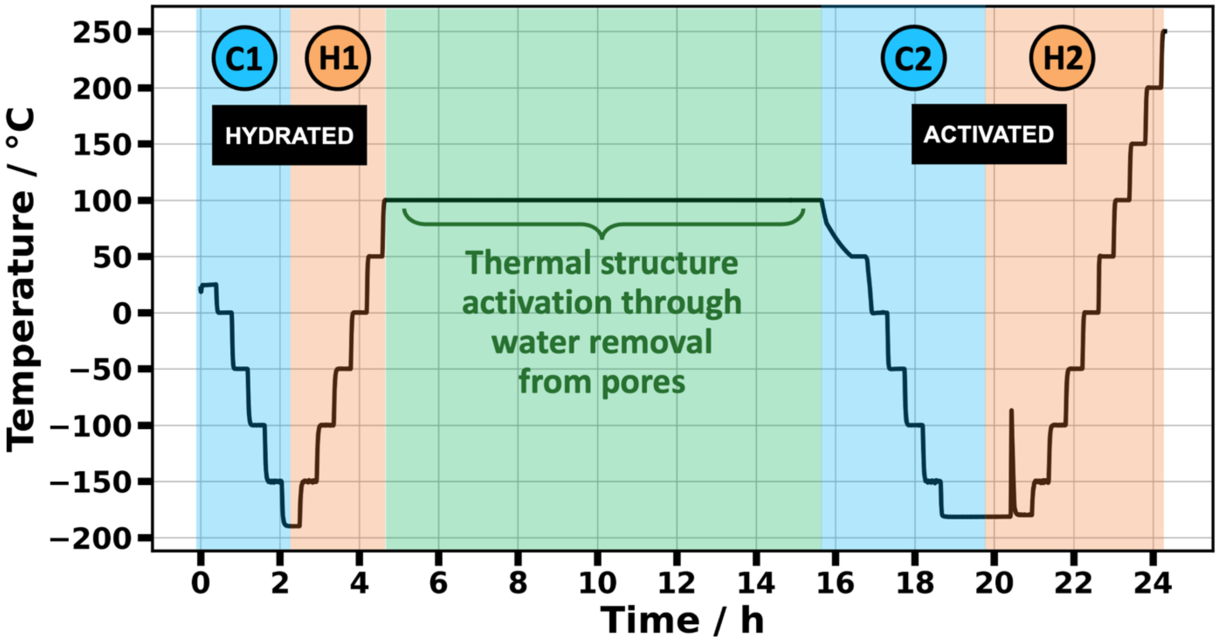

Fig. S13. Temperature program recorded by the temperature sensor used to study the thermal expansion of hydrated and activated GUT-2. Temperature-dependent powder x-ray diffraction pattern were measured at each of the set points (plateaus of constant temperature) along the cooling (C1, C2) and heating curves (H1, H2).

References

1 C. F. Macrae, P. R. Edgington, P. McCabe, E. Pidcock, G. P. Shields, R. Taylor, M. Towler and J. van de Streek, *J. Appl. Crystallogr.*, 2006, **39**, 453–457.

2 B. Lee and F. M. Richards, *J. Mol. Biol.*, 1971, **55**, 379-IN4.

3 K. Kodolitsch, A. Torvisco, T. Kamencek, M. Mazaj, E. Zojer and C. Slugovc, *Eur. J. Inorg. Chem.*, 2025, e202500032.

4 W. Massa, *Crystal Structure Determination*, Springer Berlin Heidelberg, Berlin, Heidelberg, 2004.

5 P. Müller, R. Herbst-Irmer, A. L. Spek, T. R. Schneider and M. R. Sawaya, *Crystal Structure Refinement: A Crystallographer's Guide to SHELXL*, Oxford University Press, New York, United States, 2006.

6 O. V. Dolomanov, L. J. Bourhis, R. J. Gildea, J. A. K. Howard and H. Puschmann, *J. Appl. Crystallogr.*, 2009, **42**, 339–341.

7 P. Scherrer, in *Kolloidchemie Ein Lehrbuch*, Springer Berlin Heidelberg, Berlin, Heidelberg, 1912, pp. 387–409.

8 W. A. Rachinger, *J.Sci.Instrum.*, 1948, **25**, 254.

9 T. Degen, M. Sadki, E. Bron, U. König and G. Nénert, *Powder Diffr.*, 2014, **29**, S13–S18.

10 R. Gross and A. Marx, *Festkörperphysik*, Walter de Gruyter, Berlin, 3rd edn., 2018.

11 C. Kittel, *Einführung in die Festkörperphysik*, Oldenbourg Wissenschaftsverlag, München, 15th edn., 2013.
12 F. Mouhat and F.-X. Coudert, *Phys. Rev. B*, 2014, **90**, 224104.
13 F. P. Lindner, N. Strasser, M. Schultze, S. Wieser, C. Slugovc, K. Elsayad, K. J. Koski, E. Zojer and C. Czibula, *J. Phys. Chem. Lett.*, 2025, **16**, 1213–1220.
14 A. Erba, J. K. Desmarais, S. Casassa, B. Civalleri, L. Donà, I. J. Bush, B. Searle, L. Maschio, L. Edith-Daga, A. Cossard, C. Ribaldone, E. Ascrizzi, N. L. Marana, J.-P. Flament and B. Kirtman, *J. Chem. Theory Comput.*, 2022, acs.jctc.2c00958.
15 J. E. Jaffe and A. C. Hess, *Phys. Rev. B*, 1993, **48**, 7903–7909.
16 J.-C. Tan, B. Civalleri, C.-C. Lin, L. Valenzano, R. Galvelis, P.-F. Chen, T. D. Bennett, C. Mellot-Draznieks, C. M. Zicovich-Wilson and A. K. Cheetham, *Phys. Rev. Lett.*, 2012, **108**, 095502.
17 J. Heyd, J. E. Peralta, G. E. Scuseria and R. L. Martin, *J. Chem. Phys.*, 2005, **123**, 174101.
18 J. P. Perdew, K. Burke and Y. Wang, *Phys. Rev. B*, 1996, **54**, 16533–16539.
19 S. Grimme, J. Antony, S. Ehrlich and H. Krieg, *J. Chem. Phys.*, 2010, **132**, 154104.
